# Injuries in outdoor climbing: a retrospective single-centre cohort study at a level 1 emergency department in Switzerland

**DOI:** 10.1136/bmjsem-2021-001281

**Published:** 2022-03-29

**Authors:** Chantal Selina Krieger, Doris-Viviana Vesa, Stephan Ziegenhorn, Aristomenis Konstantinos Exadaktylos, Jolanta Klukowska-Rötzler, Monika Brodmann Maeder

**Affiliations:** 1 Department of Emergency Medicine University Hospital, Inselspital University Hospital Bern, Bern, Switzerland; 2 Institute for Mountain Emergency Medicine, EURAC Research, Bolzano, Italy

**Keywords:** Rock climbing, Injuries, Sporting injuries

## Abstract

**Objectives:**

Outdoor rock climbing has become popular in recent years. However, few data have been published on climbing accidents in Switzerland, even though the Swiss Alps are a major climbing resort.

To analyse data on accidents related to outdoor climbing treated in the Emergency Department (ED) of University Hospital Bern, Switzerland.

**Methods:**

A retrospective database search for accidents related to outdoor climbing was conducted in the clinical reporting system E.care of the ED of University Hospital Bern for the period April 2012–December 2018.

**Results:**

78 patients were treated after an accident related to outdoor climbing, which accounted for 1 per 3571 (0.028%) of all ED visits during this period. Mean age was 35.8±10.4 years. 76% of patients were male. Falls were the most common mechanism of injury (64%), followed by rock or ice falling on the climber (21%). Injuries affected multiple body regions (38%) or only the lower limbs (22%). Most injuries were fractures (68%). Mean ISS was 7.5 (1–38), and grade 3 UIAA MedCom injuries were most common (45%). 11 cases of polytrauma occurred and one fatality. 44 patients needed inpatient admission. Mean duration of inpatient stay was 7 days. Mean costs per patient were 12 283 CHF.

**Conclusions:**

Accidents related to outdoor climbing accounted for a small number of patients seen in the University ED Bern. Further research should be on a nationwide basis, with collection of specific climbing data like use of a helmet and experience of climbing to inform injury prevention strategies. This should shed further light on this topic, as would a prospective study using the International Alpine Trauma Register.

**Key messages**

**What is already known on this topic**

- Rock climbing has become increasingly popular worldwide. Climbing injuries can be classified as acute traumatic injuries, atraumatic and chronic overuse injuries.

**What this study adds**

- To our knowledge, this is the first study in Switzerland to specifically examine outdoor climbing accidents in an emergency department.

**How this study might affect research, practice or policy**

- Climbing on ice is increasingly popular and this leads to new risks for injuries.

## Introduction

Rock climbing has become increasingly popular worldwide as recreational activity and competitive sport. Where previous participants were predominantly elite, dedicated climbers, the current participant pool consists of a broad spectrum of ages and levels of experience.^1^

The Swiss Alpine Club was founded in 1863 and now has approximately 160 000 members. It is one of the largest Swiss sports clubs, and many of its members are active mountaineers and climbers.^2^ According to a 2020 survey by the Swiss Federal Office of Sports, 3.5% of the Swiss population over fifteen regularly participate in climbing or mountaineering, an increase of 1.3% from 2014 to 2020.^3^ After the opening of the first climbing hall in Switzerland in 1993, the popularity of climbing has grown steadily, and there are now about fifty large climbing and boulder halls.^4^ Within the same period, the first via ferrata (fixed outdoor climbing route) was created in 1993, and today, there are more than 150 routes.^5^ Climbing is being increasingly included in school sports, physiotherapy and as training in rehabilitation.^6^ After the introduction of rock climbing to the Olympic Games in 2020, it is expected that the sport will continue to grow and expand its reputation.^7^

### Climbing disciplines

Climbing can be subdivided into indoor climbing, which takes place on special facilities on artificial walls, and outdoor climbing. Outdoor climbing can further be subdivided into ice climbing (ice axes and crampons are used to climb ice formations), bouldering (rock faces are ascended with restricted height without belaying), outdoor sports climbing (permanent or preplaced protective bolts are attached), clean climbing (bolts are attached and removed by the climber himself) and traditional mountaineering (mountain ascent).^8^ We decided to focus on all aspects of outdoor climbing for our research.

### Current studies

With increasing popularity and more participants, climbing-related injuries might be seen more frequent.^9^ In order to analyse patterns of injury in climbing, it is useful to differentiate between acute traumatic injuries, atraumatic injuries (supraphysiological loading, for example, a strain), and chronic overuse injuries.^10^ Acute injuries are caused by a specific identifiable event like a fall, whereas overuse injuries result from multiple micro traumata, where no triggering event can be named.^11^

In general, climbing is considered a relatively safe sport and the risk of injury is lower than with other popular sports like basketball or soccer.^12^ A study on the risk of injuries and fatalities found that incidences are greater for alpine and ice climbing, as the risks are more objective and external than for sport climbing, indoor climbing and bouldering.^12^ Bouldering and sports climbing are associated with lower injury rates, minor injury severity and few fatalities, irrespective of whether they are performed indoors or on natural rock outdoors.^12^ In Switzerland, an average of four climbers, and one ice climber annually died between 2000 and 2018.^13^ This number is relatively low compared with the average of 184 fatal sports accidents that occur annually in Switzerland.^13^

In a survey by an Austrian mountain rescue service, the overall mortality was low. Many climbers were uninjured, but acute traumatic injuries were often severe or fatal.^10^ Likewise the International Alpine Trauma Registry suggests that climbing accidents are generally rare but potentially fatal events.^14^ Inconsistent conclusions have been reached about the effects of age, increasing years of climbing, lead climbing, skill level and high Climbing Intensity Score, all potential risk factors for climbing accidents.^14 15^ Current studies use heterogeneous definitions, scores and methodologies, which makes interstudy comparison difficult or impossible.^16 17^ To counteract this problem, the Medical Commission of the International Mountaineering and Climbing Federation UIAA introduced classifications for analysing climbing and mountaineering accidents and illnesses in 2011, including definitions for injury location, injury classification and fatality risk.^17^

## Methods

### Settings

The Department of Emergency Medicine (ED) of the University Hospital of Bern, Switzerland, is a level 1 trauma centre with approximately 50 000 patients per year. The University Hospital of Bern is one of five University Hospitals in Switzerland and part of the Swiss trauma network. This network consists of 12 hospitals in total that are specialised and assigned for the care of seriously injured patients and provide highly specialised trauma medicine.^18^ The catchment area includes various parts of the Alps, as well as climbing gardens, viae ferratae and high ropes courses.

### Data collection

The clinical reporting system E.care (BVBA-beslotet vennootschap, Turnhout, Belgium) was searched for the German keywords ‘climbing’ and ‘boulder’. A total of 222 cases could be found during the period from 15 April 2012 to 31 December 2018. All 222 medical records were anonymised, then fully read. All patients with evidence for an outdoor climbing accident in the medical report were included. Exclusion criteria were indoor climbing accidents, incomplete information, and lack of a direct correlation between injury and climbing accident. Patients aged under 16 were treated in the Department for children and adolescents and were therefore not included in this study. Information about demographics, mechanism of injury, diagnosis, treatment and hospital stay were collected. The full cost of the care in the emergency department (ED) and the hospital stay were compiled for each case in an additional search.

The Injury Severity Score (ISS), a score based on the Abbreviated Injury Scale, was used to retrospectively classify injuries based on the diagnosis, on a scale from 0 (uninjured) to 75 (worst outcome). A score greater than 15 is defined as a polytrauma. In addition, the UIAA MedCom Score was used to classify the injuries as 0 (uninjured), 1 (mild injury), 2 (moderate injury), 3 (major injury), 4 (alive with permanent damage), 5 (death due to injury), and 6 (immediate death). Injury location was classified with the Orchard Sports Injury Classification System (OSICS) 10, as proposed by the UIAA-medical commission.^17^ Patients were divided into a rock and ice fall group and a group with other mechanisms of injury. A retrospective, descriptive data analysis (percentage and mean) was performed with Microsoft Excel.

## Results

### Demographics

Table 1 For the period from 15 April 2012 to 31 December 2018, a total of 78 patients could be included who were treated at the University Hospital of Bern due to an injury related to outdoor climbing. Between 8 and 14 patients were admitted annually, which accounts for about 1 per 3571 (0.028%) of all ED visits during that period. Age distribution was between 18 and 68 years, with a mean age of 35.8±10.4 (SD) years, median of 34 and IQR of 11.75 years. 76% (n=59) of all patients were male. Most accidents occurred during the summer (n=37; 47%). Rock and icefall accidents accounted for 0 to 5 patients annually. Over the years, injuries due to rock and ice fall were accounted for an increasing proportion of the visits to the ED. In 2018, five of nine patients were injured due to falling rock or ice.

**Table 1:** Characteristics according to mechanism of injury

Characteristics	Total (n)	(%)	Rock and ice fall (n)	(%)	Other mechanisms of injury (n)	(%)
Age in years						
18–25	12	15.38	3	16.67	9	15.00
26–35	31	39.74	7	38.89	24	40.00
36–45	21	26.92	4	22.22	17	28.33
46–55	10	12.82	3	16.67	7	11.67
>56	4	5.13	1	5.56	3	5.00
Gender						
male	59	75.64	13	72.22	46	76.67
female	19	24.36	5	27.78	14	23.33
Year						
2012	8	10.26	0	0.00	8	13.33
2013	11	14.10	2	11.11	9	15.00
2014	12	15.38	1	5.56	11	18.33
2015	10	12.82	3	16.67	7	11.67
2016	14	17.95	3	16.67	11	18.33
2017	14	17.95	4	22.22	10	16.67
2018	9	11.54	5	27.78	4	6.67

### Climbing-specific parameters

Sixty-six patients (85%) participated in outdoor rock climbing, and 12 patients (15%) were ice climbers.

#### Mechanism, severity and localisation of injury

Table 2 Eighteen (23%) patients were included in the rock and ice fall group. The mechanisms of injury in the other patients included fall, hit and slip, distortion and others (n=60; 77%). Direct falls accounted for 64% (n=50) of all injuries, followed by injuries provoked by falling rock or ice with 23% (n=18), hit and slip (n=4), distortions (n=4), and others (n=2). In three cases, rock and ice fall was followed by a fall of the patient.

**Table 2:** Analysis by mechanism of injury

Characteristics	Total (n=78)	(%)	Rock and ice fall (n=18)	(%)	Other mechanism of injury (n=60)	(%)
Fall height						
No fall/ fall height not registered	31	39.74	15	83.34	16	26.67
1–5 m	11	14.10	1	5.56	10	16.67
6–10 m	21	26.92	1	5.56	20	33.33
11–20	6	7.67	0	0.00	6	10.00
21–30 m	5	6.41	1	5.56	4	6.67
>30 m	4	5.13	0	0.00	4	6.67
Injury location						
Multiple body regions*	30	38.46	2	11.11	28	46.67
Isolated head and neck	16	20.51	9	50.00	7	11.67
Isolated upper limbs	9	11.54	3	16.67	6	10.00
Isolated trunk	6	7.69	2	11.11	4	6.67
Isolated lower limbs	17	21.79	2	11.11	15	25.00
Total	78	100	18	100	60	100
ISS mean	7.52		6.11		7.95	
UIAA MedCom Score						
1	3	3.85	2	11.11	1	1.67
2	31	39.74	6	33.33	25	41.67
3	35	44.87	8	44.44	27	45.00
4	8	10.26	2	11.11	6	10.00
5	1	1.28	0	0.00	1	1.67
Diagnosis						
Fracture	53	67.95	10	55.56	43	71.67
Laceration	29	37.18	8	44.44	21	35.00
Soft tissue†	20	25.64	8	44.44	12	20.00
Concussion	19	24.36	3	16.67	16	26.67
Sprain, strain	9	11.54	1	5.56	8	13.33
Contusion/laceration of organs	8	10.26	2	11.11	6	10.00
Pneumothorax	6	7.69	1	5.56	5	8.33
Intracranial haemorrhage	6	7.69	2	11.11	4	6.67
Luxation, subluxation	3	3.85	0	0.00	3	5.00
Others	8	10.26	3	16.67	5	8.33

*Superficial wounds like bruises, haematomas or abrasions were not counted as multiple injuries.

†Soft tissue including abrasions, haematomas and contusions.

ISS, Injury Severity Score.

**Figure 1:**
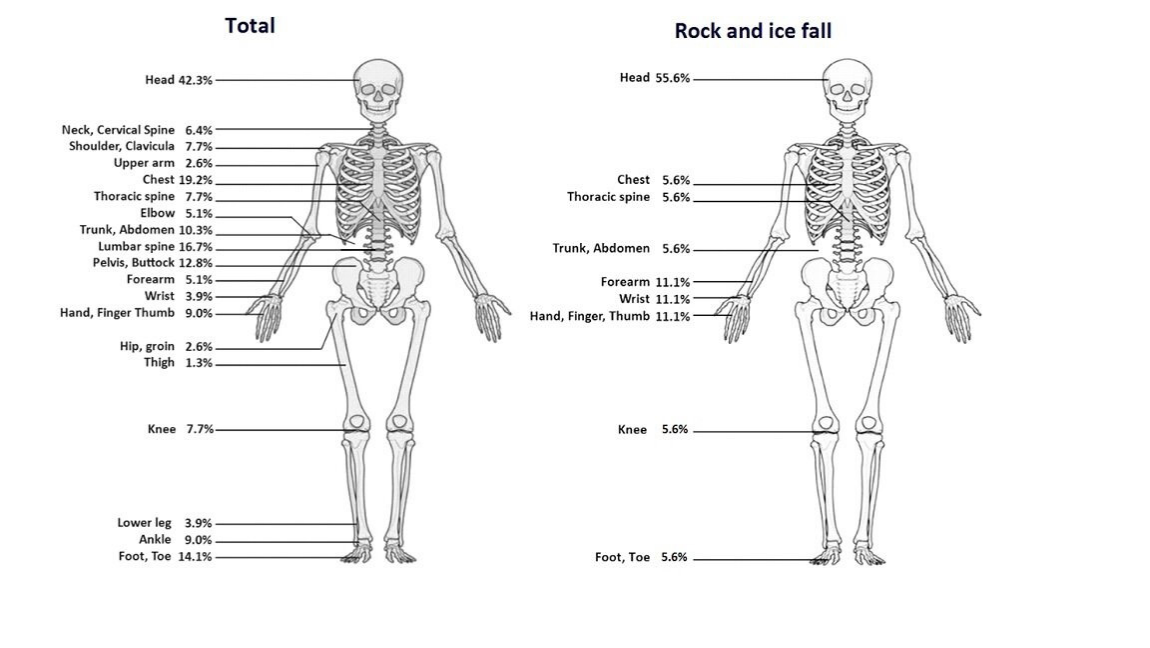
Anatomical sites according to Orchard Sports Injury Classification System 10 and mechanism of injury.

Fall height was registered in 47 cases (60%) and varied from 1 m to 50 m, with most falls occurring between 1–10 metres (41%). In seven records, information about the fall height was missing. Through the anatomical site analysis with OSICS 10, we found that head injuries were most frequent (n=33; 42%), followed by injuries to the chest (n=15; 19%), lumbar spine (n=13; 17%) and foot/toe (n=11; 14%) (figure 1). In this analysis, multiple and isolated injuries were included. When it is divided, in 38% (n=30) of injuries, multiple body regions were affected, the rest were isolated lower limbs (n=17), head and neck (n=16), upper limbs (n=9) and trunk injuries (n=6).

**Figure 2:**
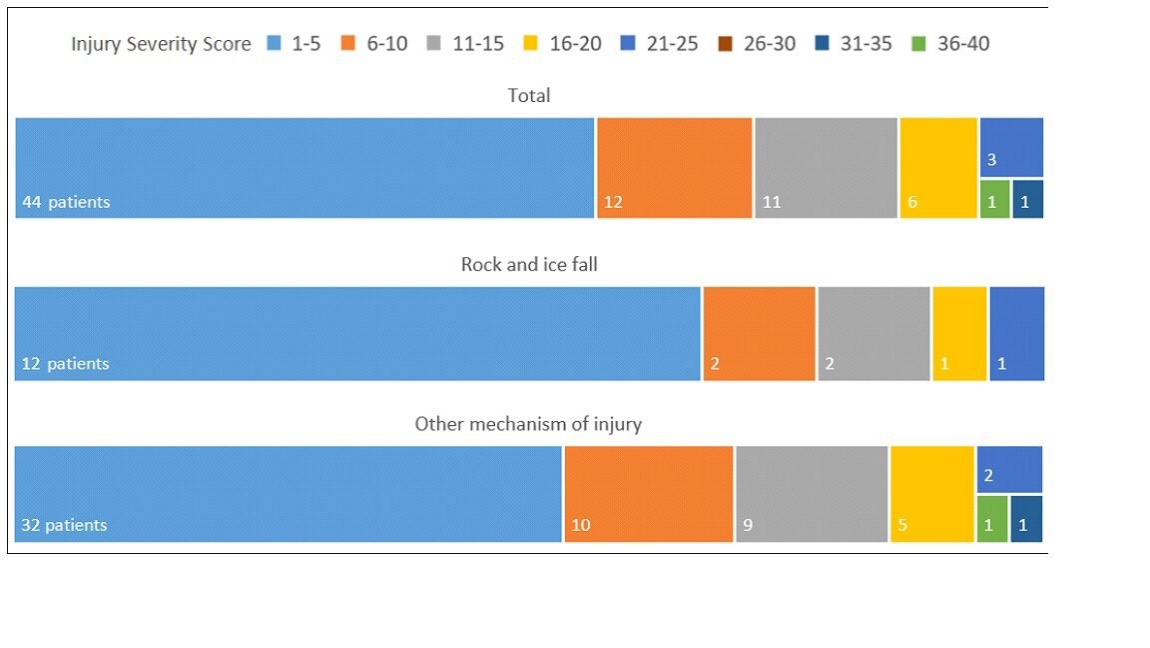
Injury Severity Score by mechanism of injury.

Injury severity as based on the ISS had an overall mean of 7.5 points (range 1–38) (figure 2). Patients injured through rock and ice fall had a mean ISS Score that was 1.9 points lower (6.1 and 8.0) than other patients. Polytrauma occurred in 14% of patients (n=11). All patients with polytrauma were male. The patient with the highest ISS (ISS 38) died 2 days after hospital admission. He suffered from haemorrhagic shock, multiple fractures and severe traumatic brain injury (TBI) with multiple intracranial haemorrhages and shearing injuries. Due to the very poor prognosis, therapy was discontinued. No patient died in the ED.

Severity of injury as based on the UIAA MedCom Score was similarly distributed in all groups for grade 2, 3 and 4 injuries, with grade 3 injuries being the most common with 45% (n=35). Climbers injured by rock and ice fall had more grade 1 injuries (11%) than in the other subgroups (1.7%). There was one grade 5 injury (fatal). No grade 0 or grade 6 injuries were registered.

Fractures were the most common type of injury (n=53; 68%), and the trunk was most often affected (n=27; 35%), followed by head and neck (n=20; 26%) and lower limbs (n=14; 18%). Lacerations (n=29; 37%), soft tissue injuries (n=20; 26%), were among the most common injuries. TBI accounted for 24% of reported injuries with 14 mild, 2 moderate and 3 severe brain injuries. Intracranial haemorrhage occurred in six patients (8%).

#### Hospitalisation and costs

Table 3 Thirty-four patients (44%) were treated as outpatients, 44 patients (56%) were hospitalised. Mean duration of inpatient stay was 7 days, with a range between 1 and 19 days. Surgery was indicated in 33 patients.

**Table 3:** Hospitalisation and costs by mechanism of injury

Characteristics	Total (n)	(%)	Rock and ice fall (n)	(%)	Other mechanisms of injury(n)	(%)
Use of the resuscitation room	37	47.44	7	38.89	30	50.00
Procedure						
outpatient treatment	34	43.59	8	44.44	26	43.33
hospitalisation	44	56.41	10	55.56	34	56.67
Duration of hospitalisation in days						
0	34	43.59	8	44.44	26	43.33
1–5	24	30.77	7	38.89	17	28.33
6–10	9	11.54	1	5.56	8	13.33
11–15	7	8.97	2	11.11	5	8.33
16–20	4	5.13	0	0.00	4	6.67
Therapy						
no surgery	45	57.69	11	61.11	34	56.67
surgery	33	42.31	7	38.89	26	43.33
Inability to work						
none	14	17.95	5	27.78	9	15.00
certificate of incapacity for work	46	58.94	12	66.67	34	56.67
no information / transferred	17	21.79	1	5.56	16	26.67
Costs in CHF						
1–10 000	51	65.38	13	72.22	38	63.33
10 001–20 000	9	11.54	3	16.67	6	10.00
20 001–30 000	7	8.97	0	0.00	7	11.67
30'001–40 000	5	6.41	0	0.00	5	8.33
>40 000	6	7.69	2	11.11	4	6.67
mean	12 283		10 894		12 700	
Total	958 105	100.00	196 105	20.47	762 000	79.53

CHF, Swiss franc currency.

The total costs for all 78 patients amounted to of 958 105 CHF, which results in a mean of 12 283 CHF per patient (range 98–61 942 CHF). The mean costs for outpatient treatment were 1328 CHF, in comparison with 20 746 CHF for inpatient treatment. Costs were in a range from 1 to 5000 CHF in 38 cases, from 5000 to 10 000 CHF in 13 cases, and >10 000 CHF in 27 cases. Six patients caused costs of over 40 000 CHF, resulting in 35% of all costs. These were patients with multiple injuries, an UIAA score between three and five and five patients suffered from polytrauma.

Information about inability to work was provided in 67 patient records. Forty-six patients (59%) received a certificate of incapacity for work after the climbing accident.

## Discussion

In this retrospective cohort study of outdoor climbing accidents presenting at the ED of the University Hospital in Bern between 2012 and 2018, 78 patients were included. They accounted for 1 per 3571 of all ED patients treated in the same period. This is in line with a study by Buzzacott *et al* who found that 0.03% of injuries treated in US EDs were climbing related.^19^ Despite the increasing number of climbing enthusiasts, there was no evident increase over the years in the number of patients treated in the ED of University Hospital in Bern for outdoor climbing accidents. This contrasts with two comprehensive analyses in US EDs, which demonstrated an increase in consultations due to climbing-related accidents over the years.^1 19^ As we only collected data from the University Hospital ED Bern, an increase in climbing related accidents in out-patient clinic and general practitioner is possible. Further, data would need to be collected over a longer period, to assess a trend. The increasing number of participants could also be primarily in indoor climbing, as it probably needs less prior knowledge and could be accessed more easily.

Due to the large heterogeneity in the literature, data on the incidence of climbing accidents vary widely.^14^ In line with the publications that state that climbing accidents mostly affect a young and healthy population, our cohort was a young, predominantly male patient population with a mean age of 35.8 years and a normal body mass index.^14^ They needed inpatient treatment in more than half of the cases. As the University Hospital in Bern is the only level 1 trauma centre in the region, and patients with less severe climbing injuries may also be treated in other hospitals or visit a general practitioner, we might have overestimated the severity of these accidents.

### Mechanism of injury

According to various studies, falls are the most common mechanism of injury in outdoor rock climbing and ice climbing, and cause between 55% and 77.5% of all accidents.^1 14 19–24^ This is consistent with our result of 64.1%. We found that a surprisingly high proportion of injuries (23.1%) were caused by rock or ice falling on the victim. In contrast, retrospective analysis from American EDs identified only a few such injuries (3% or 6.4% of all injuries). Both studies analysed data from the National Electronic Injury Surveillance System registry, a register of ED presentations collected from around 100 hospitals in the USA.^1 19^ The reason for this discrepancy could be that the cited studies also included indoor climbing activities. Presumably less experience and less risk averse people could participate in indoor climbing, as the ropes and harnesses are a requirement and therefore the injury patterns may differ.

The more frequent rock and ice falls in our catchment area might be due to climate change and melting of the permafrost. As we only have a limited sample of patients, further investigation is required. Nearly all injuries were caused by an acute trauma. We only registered one injury due to overexertion: A medianus lesion was suspected due to the heavy load while climbing. Nelson *et al* and Buzzacott *et al* registered between 3% and 15% of overexertion injuries.^1 19^

Classification based on case narratives may be subjective and therefore another reason for the difference in distribution of mechanisms of injury.^19^ For instance, in our study three patients additionally fell after the ice/rock fall, and these were classified as part of the ice and rock fall mechanism, whereas they could also be classified as falls.

Due to a lack of information, the fall height was difficult to analyse since it was presumably based mainly on an estimation. For example, five patients reported falling more than 30 metres and in these patients, severity of injury varied greatly between ISS 1–38 and UIAA MedCom Score 2–5. Furthermore, in most cases it remained unclear whether the fall was decelerated by a rope and how steep the terrain was.

In non-impact acute injuries and chronic overuse injuries, the upper limbs seem to be mostly affected, especially the fingers.^25–28^ Injuries in alpine climbing mostly occur through falls and affect the lower extremity.^16^ With 46.3%–47%, the lower extremity was mostly affected in two analysis of patient data from American ED.^1 19^ In another analysis of American EDs and similarly to our results, Forrester *et al* found that multiple body regions were often affected.^9^ Rugg *et al* reported that in fatal accidents multiple body injuries were most frequent and that injuries to the lower extremities predominated in severe accidents.^10^ Neuhof *et al*^29^ found that injuries in sport climbing were equally distributed between upper and lower limbs.

### Severity of injury

Various studies have analysed the severity of injury using the UIAA MedCom score (Union Internationale des Associations d'Alpinisme Score).^10 19 29–31^ It is difficult to compare the studies, due to the differences in study design. In a study conducted during an ice climbing festival, most injuries were mild (UIAA 1).^30^ Most injuries were moderate (grade 2) in a case series in a German hospital with elective outpatient and emergency treatment, and in an internet survey of sport climbing injuries.^29 32^ In a study in an indoor climbing hall, grade 1 injuries were excluded, but grades 2 and 3 were about equally common (15 grade 2 and 13 grade 3 injuries) hall.^31^ In an analysis of rescue services in Austria, most patients (n=1469) were uninjured, followed by grade 3–5 injuries (n=703).^10^ In contrast to our results, Buzzacott *et al* found more moderate injuries and fewer UIAA grade 3–5 injuries in US EDs.^19^ Likewise, the number of patients requiring inpatient treatment in our cohort was high at 56.4%, in contrast to other studies in EDs, in which 8%–11.3% of patients were hospitalised.^1 9 19^ This could be explained by the fact that indoor climbing accidents were not included in our study and are presumed to lead to injuries of minor severity and with fewer fatalities.^12^ As in our analysis, fractures accounted for the majority of injuries in patients treated in EDs.^1 9 19^ During the 6-year period, we only identified one inpatient fatality, so we suggest that climbing related deaths mostly occurred at the site of injury, and these cases, were not included in our study. This is in accordance with Forrester *et al*.^9^ Injury prevention is essential and should include careful planning, appropriate equipment, checking weather and environmental conditions as well as checking the anchors and the partners’ equipment.^33^

### Costs

To our knowledge, the multicentre, retrospective ED-analysis by Forrester *et al*, is the only study that has reported costs for climbing related hospital treatment.^9^ With a median of US$65 000 for inpatient treatment, their costs were much higher than ours, where mean costs were 1328 CHF (Swiss franc) for outpatient treatment and 20 746 CHF for inpatient treatment. This must be interpreted in the context of the different cost structures in the different countries. Inability to work was frequent and caused additional financial burdens that were not included in our analysis.

### Limitations

As the University Hospital Bern is a level 1 trauma centre and part of the Swiss trauma network, it is more likely to treat severe accidents, which might cause a selection bias. Furthermore, as this was a single-centre study, only accidents in the catchment area of the trauma centre were registered, whereas a nationwide analysis could provide more information about prevalence and injury trends. A systematic data collection on climbing specific parameters, like climbing discipline, belaying technique, climbing level and use of a helmet, could not be performed due to the lack of structured data. Standardised questionnaires for climbing parameters could be completed in the ED and would enable more accurate analysis.

## Conclusion

Even though climbing is increasingly popular and there are more hobby climbers, we found no evidence for an increase in the incidence of accidents from outdoor climbing. In our cohort, hospitalisation rates were high, and injuries provoked by falling rock or ice were frequent and accounted for a higher proportion of visits to the ED over the years.

## Data Availability

Data sharing not applicable as no datasets generated and/or analysed for this study.
